# A health economics study of long-acting injectable once-monthly paliperidone palmitate in schizophrenia: a one-year mirror-image study in China

**DOI:** 10.1186/s12888-022-03728-2

**Published:** 2022-02-08

**Authors:** Jie Liu, Qian Wang, Lei Su, Limin Yang, Lianyong Zou, Ludong Bai

**Affiliations:** Shandong Mental Health Center, No.49 Wenhua East Road, 250014 Jinan, Shandong, People’s Republic of China

**Keywords:** Schizophrenia, Direct costs, Indirect costs, Health economics, Once-monthly paliperidone palmitate

## Abstract

Schizophrenia is ranked among the top 25 leading causes of disability worldwide in 2013 which resulting in social and economic burden. By observing patients with schizophrenia one year before and after switching from oral antipsychotics (OAPs) to once-monthly paliperidone palmitate (PP1M), we can better understand the change of total costs in schizophrenic patients, including direct costs and indirect costs, after switching treatment patterns.

A total of 100 schizophrenic (ICD-10) patients from Shandong Mental Health Center were collected from December 2016 to June 2019. Treatment modalities, health care resource utilization and costs were compared before and after switching directly from oral antipsychotics to PP1M.

Of the 82 patients included in the main analyses, treatment with PP1M resulted in an increase in direct costs of 31.92% (P < 0.01), an increase in medicine costs of approximately 142% (P < 0.01), and a reduction in hospital costs of 68.15% (P > 0.05). There was no significant increase in total costs (P = 0.25), while 31.92% increase in direct costs (P < 0.01), and 35.62% decrease in indirect costs (P < 0.01) after conversion to PP1M. Compared with before administration of PP1M, patients with ≥ 1 inpatient stay in 1 year Pre-PP1M treatment with OAPs (n = 32) had a 20.16% decrease in direct costs (P < 0.01), a 144% increase in medicine costs (P < 0.01), and a significant 72.02% decrease in hospital costs (P < 0.01). The observed reduction in the number of hospitalizations (t = 2.56, P ≤ 0.01) and inpatient stays (t = 1.73, P < 0.05) and after transition to PP1M resulted in a reduction in hospitalization costs (P < 0.01).

Switching from OAPs to PP1M decreased the household workforce burden without increasing clinical healthcare costs. Direct costs were significantly reduced in patients with ≥ 1 inpatient stay in 1 year pre-PP1M treatment with OAPs after the switch, which decreased by improving adherence to therapy and reducing the number and length of hospital stays, suggesting that those patients may benefit after switching to PP1M.

## Introduction

Schizophrenia is a remitting and relapsing psychiatric disorder, characterized by profound disruptions in thinking, language, perception, and self-perception [1]. Schizophrenia is ranked among the top 25 leading causes of disability worldwide in 2013 [2]. The average age of onset of the disease is between 15 and 35 years, usually in late adolescence or in the early 20 s in men and later in women [1]. Despite its low prevalence, its social and economic burden remains substantial, not only for patients, but also for families, and more broadly for society. Economic burden studies often incorporate both direct and indirect costs. Direct costs are costs associated with hospital inpatient treatment, prescription medications, long-term institutional care, and treatment costs associated with psychological and physical complications [3]. Indirect costs are defined as morbidity, premature mortality, and productivity losses related to informal care provided by caregivers with schizophrenia [4–7]. There are two main reasons causing the high economic burden of schizophrenia. First, non-adherence to antipsychotic use is a serious contributor to the economic burden of schizophrenia. Approximately one-third of patients with schizophrenia have poor adherence to treatment with OAPs [2]. Non-adherence to treatment can hinder the success of treatment and may lead to poor clinical outcomes and a higher risk of relapse and rehospitalization [8–11], which is also associated with increased direct cost. Second, the impairment of physical function and quality of life in schizophrenia, which often leads many nursing responsibilities for family members [12] and increases indirect burden. In fact, one study has shown that between 50 and 80% of schizophrenic patients live closely with family members [12]. Furthermore, there is a third type of costs is referred to as an intangible fee. These are associated with decreased quality of life of patients, families, and caregivers due to other factors such as pain or distress [13]. But these costs are extremely difficult to quantify and are therefore often ignored in economic research [14].

In addition, long-acting injectable antipsychotics (LAIs) have been shown to increase medication adherence [15, 16], reduce relapse and rehospitalization [16–18], and are even more cost-effective than OAPs. Although there is much strong evidence recommending the use of LAIs, it remains underutilized in clinical practice [19, 20] due to the so-called high costs [21]. Paliperidone palmitate is an antipsychotic in the form of a long-acting injectable formulation approved for the treatment of schizophrenia and schizoaffective disorders [22]. PP1M is intended for once-monthly intramuscular injection and it does not require any oral supplementation [23, 24], After injection, PP1M slowly dissolves owing to its extremely low water solubility, and absorbed into the systemic circulation [25]. There are currently several second-generation antipsychotic long-acting injections approved in China as options. However, PP1M was the once-monthly injectable second-generation antipsychotic approved in our hospital during our study. This study was conducted because we believe the following questions remain: which clinical treatment is more economical between OAPs and LAIs. Based on which treatment is more cost-effective, the goal of our study is understanding the change of total costs in schizophrenic patients, including direct costs and indirect costs, after switching treatment from PP1M to OAPs. We hope to provide data support for the efficient and reasonable utilization of social health resources and for the reduction of social medical treatment costs.

## Methods

### Subjects

A total of 100 patients with schizophrenia (ICD-10) who could be followed for at least one year in Shandong Mental Health Center were collected from December 2016 to June 2019. Patients with ≥1 hospitalization experience in one year before mirror point were included in the statistical analysis. The inclusion criteria were as follows: (a) between 18 and 65 years old; (b) subjects with the course of the disease ≥1 year; (c) subjects were in stable condition (Positive and Negative Syndrome Scale (PANSS) total score < 70 or Clinical Global Impression-Severity (CGI-S) score of ≤3 (mild ill); (d) subjects with at least one illness exacerbation or hospitalization (PANSS score ≥70) in the year prior to enrollment; (e) the date of the first observation of a diagnosis of schizophrenia during this period was identified as the initial date; (f) subjects received paliperidone palmitate at their own will; (g) taking antipsychotic drugs ≥6 months within in one year before mirror point. The exclusion criteria were as follows: (a) pregnant or lactating women; (b) alcoholism or substance abuse; (c) patients with severe physical diseases and organic brain disease;(d) patients who could not be followed for least 1 year; (e) patients with PP1M no less than 4 times/year. We obtained data from hospital information management system. The study protocol was reviewed and approved by Ethical Committee (2017R22).

### Design and Variables

**Fig. 1:**
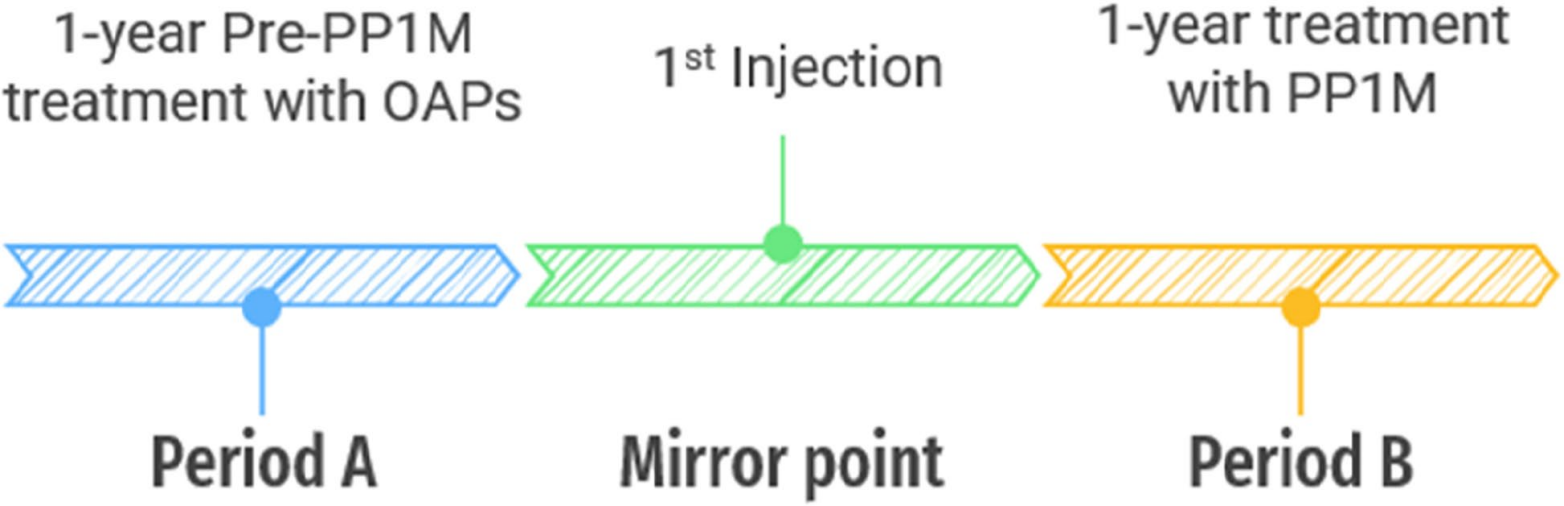
Disposition of models and periods

This was a mirror-image study. The day on which PP1M commenced was set as the “mirror point”, as show in Table 1; Fig. 1. The study compared the number of days of health care use and costs during the 1-year period before and after PP1M implementation between December 2016 and June 2019.

**Table 1:** Disposition of models and periods

1 years treatment with oral antipsychotic	1st injection	1 years treatment with paliperidone pamitate
Period A	Mirror point	Period B

Until now, direct costs were obtained through the databases. At present, objective indirect cost assessment tools are lacking. Through literature review,indirect cost is mainly evaluated by purely descriptive method or by the author’s own design of burden questionnaire,which was quantified in monetary form or in days of loss [26, 27]. In this study, we designed the economic burden scale by referring to relevant literature [23, 24]. The Economic Burden Scale assessed economic indicators where direct costs and indirect costs were quantified. Direct costs including medication and hospital costs were obtained by hospital information management system. Indirect costs included productivity costs which quantified by patient productivity loss due to psychiatric illness and caregiver care costs which quantified by caregiver loss due to patient care.

### Statistical methods

Statistical analyses were performed using 23.0 SPSS software. Demographic characteristic including age, gender and mean duration of disease were described at baseline. Means, medians, and standard deviations (SDs) were used for continuous variables with T-test, while counts and percentages were used for categorical variables. Pair Wilcoxon signed-rank tests were performed to compare differences in direct (indirect)and the total costs between the pre/post-PP1M periods in this 1-year mirror-image study. Pair T tests were performed to compare differences in number of hospitalization and inpatient stays during the period before and after PP1M.

## Results

 A total of 100 patients with schizophrenia who transitioned from oral risperidone or oral paliperidone to PP1M were included in the study. 18 patients fell off, including that 7 patients lost contact, 2 patients became agitated and could not tolerate side effects, and 9 patients were unwilling to continue use PP1M for personal reasons. Patients with ≥1 hospitalization experience in one year before mirror point were 32.

### Demographic Before Transition to PP1M

Of the 82 patients included in the main analyses, the average age was 32.2 (SD=1.2) years old and the mean duration of disease was 57.5 months (SD=40.6)53.66% of the patients were female and the mean CGI-S score was 2.83 (SD=0.39).

### Direct Costs Before and After Transition to PP1M

Compared with pre-PP1M, medicine costs increased significantly post-PP1M (P < 0.01), while the hospitalization costs decreased 68.15% from ¥9,155 to ¥2,915 ( P > 0.05). To sum up, direct costs increased significantly from ¥17,457 to ¥23,030 (P < 0.01), as shown in Table 2.

**Table 2:** Wilcoxon sign-rank test was used to test the difference between pre-PP1M and Post-PP1M

	Medicine costs	Hospitalization costs	Direct costs
pre-PP1M (¥)	8302 ± 4156	9155 ± 18816	17457 ± 17414
Post-PP1M (¥)	20121 ± 4580	2915 ± 8466	23030 ± 8648
Difference	142%	-68.15%	31.92%
P	< 0.01	0.074	< 0.01

### Direct Costs for Patients with ≥1 Hospitalization in 1 Year Pre-PP1M Treatment with OAP

Compared with pre-PP1M, medicine costs in inpatient stay costs increased post-PP1M (P < 0.01), and the hospital costs decreased significantly from ¥25,656 to ¥7,179 (P < 0.01). An obvious decrease in direct costs of ≥1 hospitalization in 1 year pre-PP1M treatment with OAPs were observed (P < 0.01), as shown in Table 3.

**Table 3:** Wilcoxon sign-rank test was used to test the difference between pre-PP1M and Post-PP1M. Comparison of Direct Cost for patients with ≥ 1 Inpatient Stay in 1 Year Pre-PP1M Treatment with OAPs

	Medicine costs	Hospitalization costs	Direct costs
pre-PP1M (¥)	8992±3414	25,656±18,756	34,700±18,044
Post-PP1M (¥)	21,961±5492	7179±10,552	27,705±8266
Difference	144%	-72.02%	-20.16%
P	< 0.01	< 0.01	< 0.01

### Inpatient Stays and Number of Hospitalizations after Transition to PP1M

After transition to PP1M, The number of hospitalizations(t =1.04, P > 0.05) and length of inpatient stay(t =1.73, P > 0.05) did not decrease significantly. Inpatient length of stay and number of hospitalizations (Table 4). Furthermore, as shown in Table 5. For patients ≥1 hospitalization in period A, the number of hospitalizations decreased (t =2.56, P < 0.05)and inpatient stays (t =1.73, P < 0.05) also decreased from pre- to post-PP1M period, difference between the two period has statistical significance.

**Table 4:** Comparison of Length of Stay and Number of Hospitalizations After Conversion to PP1M

	Number of hospitalizations	Inpatient stays (days)
pre-PP1M	0.67 ± 0.96	52.80 ± 60.54
Post-PP1M	0.22 ± 0.42	16.33 ± 29.48
T	2.56	1.73
P	≤ 0.01	<0.05

**Table 5:** Comparison of Length of Inpatient Stay and Number of Hospitalizations After Switching to PP1M in Patients with ≥ 1 Inpatient Stay in Period A

	Number of hospitalizations	Inpatient stays (days)
pre-PP1M	0.67±0.96	52.80±60.54
Post-PP1M	0.22±0.42	16.33±29.48
T	2.56	1.73
P	≤ 0.01	< 0.05

### Indirect Costs Before and After Transition to PP1M

Compared with pre-PP1M, productivity costs (P ≤ 0.01) and caregiver care costs (P ≤0.01) reduced post-PP1M. Consequently, indirect costs also significantly reduced (P < 0.01) (Table 6).

**Table 6:** Wilcoxon sign-rank test was used to test the difference between pre-PP1M and Post-PP1M. Comparison of Indirect Costs Before and After Transition to PP1M

	Caregiver care costs	Productivity costs	Indirect costs
pre-PP1M (¥)	3830±3260	11,767±8133	15,967±10,128
Post-PP1M (¥)	2141±4044	8137±8150	10,279±11,878
Difference	-44.10%	-30.85%	-35.62%
P	≤ 0.01	≤ 0.01	< 0.01

### Total Costs Before and After Transition to PP1M

The direct costs of the patients increased (P < 0.01), while the indirect costs decreased significantly (P < 0.05) after the switch to PP1M, by the comparison of the pre-PP1M period. Overall, the total costs to patients was no significant increased (P > 0.05), as shown in Table 7. Furthermore, as shown in Table 8. For patients ≥1 hospitalization in 1 year Pre-PP1M treatment with OAPs, an obvious decrease in indirect costs (P < 0.01)and direct costs (P < 0.01)during the transition to PP1M were observed, the total costs decreased 27% after switching to PP1M, but there was not statistically significant(P > 0.05).

**Table 7:** Wilcoxon sign-rank test was used to test the difference between pre-PP1M and Post-PP1M. Comparison of Direct Costs, Indirect Costs and Total Costs Before and After Transition to PP1M

	Direct costs	Indirect costs	Total costs
pre-PP1M (¥)	17,457±17,414	15,967±10,128	33,095±21,984
Post-PP1M (¥)	23,030±8648	10,279±11,878	33,309±15,254
Difference	31.92	-35.62%	0.64%
P	< 0.01	< 0.01	0.25

**Table 8:** Wilcoxon sign-rank test was used to test the difference between pre-PP1M and Post-PP1M For Patients with ≥ 1 Inpatient Stay in Period A , Comparison of Direct Cost, Indirect Costs and Total Costs Before and After Transition to PP1M

	Direct costs	Indirect costs	Total costs
pre-PP1M (¥)	34,700±18,044	21,718±11,246	56,418±28,020
Post-PP1M (¥)	27,705±8266	15,023±10,875	42,728±15,307
Difference	-20.16	-30.83%	-24.27%
P	< 0.01	< 0.01	0.478

## Discussion

Nowadays, oral antipsychotics remain the first-line pharmacologic treatment option for patients with schizophrenia. However, a high discontinuation rate is a known problem with oral antipsychotic treatment [28]. LAIs are generally indicated in patients who are considered non-adherent to oral therapy. In this mirror-image study, after switching from OAPs to PP1M, the number of hospitalizations (t =1.04, P > 0.05) and length of inpatient stay (t =1.73, P > 0.05) did not decrease significantly. The other study reported some different finding [29]. In this Germany mirror-image design [29], 119 patients with schizophrenia and schizoaffective disorder who switched to risperidone long acting injection (RLAI), after 12 and 18 months of RLAI treatment, the mean reduction of inpatient care was 27.4 and 38.4 days per patient, respectively. The different results may be related to the different subjects selected in the two studies. The patients selected in our study were in stable condition and took OAPs for more than six months before the mirror point. While, for patients ≥ 1 hospitalization in 1 year, using OAPs for Pre-PP1M treatment, there were not only fewer number of hospitalizations but also fewer days spent in an inpatient setting. The results of this study are consistent with those of others [20, 21]. More importantly, in addition to improvements in adherence, changes in pharmacoeconomics were also found after switching to PP1M. For the treatment of schizophrenia, most of the treatment costs do come from hospitalization [30, 31].We found that medicine costs increased, while hospital costs had no significant reduction after switch from PP1M to OAPs, resulting in an increase in direct costs (¥17,457 *versus* ¥23,030, P < 0.01). However, patients who have had ≥1 hospitalization experience in the year before the mirror point will have different consequences. Surprisingly, the decline in hospital costs could completely offset the increase in medicine costs, resulting a decrease in direct costs (¥34,700 *versus* ¥27,705, P < 0.01). We also found the same results with other research [32]. In a mirror study conducted in South Korea [32], 1272 Korean schizophrenics who switched from OAPs to PP1M were followed for 1 year: the direct costs of outpatients increased by $1497; for inpatients, direct costs was reduced by approximately $1,220. We found that the use of PP1M can partially reduce the economic burden of the disease for patients with ≥1 hospitalization experience before mirror point. Because these patients had less number of hospitalizations and length of stay. In a real-world observational study [33], the use of LAIs therapy was associated with fewer hospitalizations and fewer hospital days, which resulted in a significant reduction in monthly hospital costs ($4,007 for LAIs and $8,769 for OAPs cohort). Patients with ≥ 1 inpatient stay pre-PP1M transition may particularly benefit from switching from OAPs to PP1M, since it improves adherence and decreases the length of stay, thereby significantly reducing direct costs.

At the same time, schizophrenia patients affected by the mental symptom caused high productivity loss, and resulted in high productivity losses to patients and their families. In addition, the burden of schizophrenia, in addition to direct medicine costs also had a profound impact on caregivers and patient families [34]. Currently, few studies have observed changes in indirect costs of patients with schizophrenia from OAP to PP1M. This study found that indirect costs (productivity loss and informal care costs provided by caregivers) were effectively reduced when patients were switched from OAPs to PP1M (P ≤0.01). In the meanwhile, the quality of life of caregivers may also be improved. In summary, this study found that after the transition from OAPs to PP1M, direct costs have increased while indirect costs have decreased, resulting in a further reduction in no difference in total costs (after from OAPs transition to PP1M.

In contrast to prior studies that included a comparison of OAPs to PP1M using a cohort design, this study used a mirror-image design to compare patients before and after the initiation of PP1M. The mirror-image design is a study that compare the results before and after the medication changes (from OAPs to PP1M). The focus here is to compare the economic results of dressing changes for patients with schizophrenia before and after. There are not many studies in this direction, and previous literature review studies have focused on summarizing direct costs associated with schizophrenia. There has been a lack of emphasis on indirect costs estimation. Furthermore, China has no similar results at present. Therefore, this study focuses on the economic burden of direct and indirect costs when Chinese patients with schizophrenia switch from OAP to PP1M.

However, the finding of this study should be interpreted in the context of some limitations. First, part of our database (indirect costs) was based on patient-provided data, and its accuracy cannot be guaranteed. Second, the sample size was relatively small, and the data may not be representative of the entire Chinese population. Therefore, in the follow-up study, we will further cooperate with other regions in China to carry out follow-up research and evaluation.

## Conclusions

Comparing patients one year before and after switching from OAPs to PP1M, the direct costs increased and the indirect costs decreased, leading to a smaller difference in total costs after switching to PP1M. This suggests that patients switching from OAPs to PP1M can improve patient productivity, reduce caregiver burden and can not increase the costs of health care in the clinic. For patients with ≥1 inpatient stay one year prior to PP1M, the hospital costs decreased which were associated with the reduction of number of hospitalization and inpatient stays, and the medicine costs increased which was fully offset by lower hospital costs after switching from OAPs to PP1M. The reduction of direct costs suggests that patients with hospitalization experience obtain greater economic benefits by changing from OAPs to PP1M.

## Data Availability

All data generated or analyzed during this study are included in this published article. Data are available on request from the corresponding author.

## Abbreviations

OAPs: oral antipsychiotics
LAIs: long-acting injectable antipsychotics
PP1M: once-monthly paliperidone palmitate
OAPs: oral antipsychotics
ICD-10: international classification of diseases, tenth revison
CGI-S: clinical global impressions scale
PANSS: positive and negative syndrome scale
SD: standard deviation
RLAI: risperidone long acting injection
